# Supplementary figures and images for: Beyond accuracy: Quantifying the reliability of multiple instance learning for whole slide image classification
Source: PLoS One. 2025 Dec 5;20(12):e0337261. doi: 10.1371/journal.pone.0337261 (PMC12680235; doi:10.1371/journal.pone.0337261)

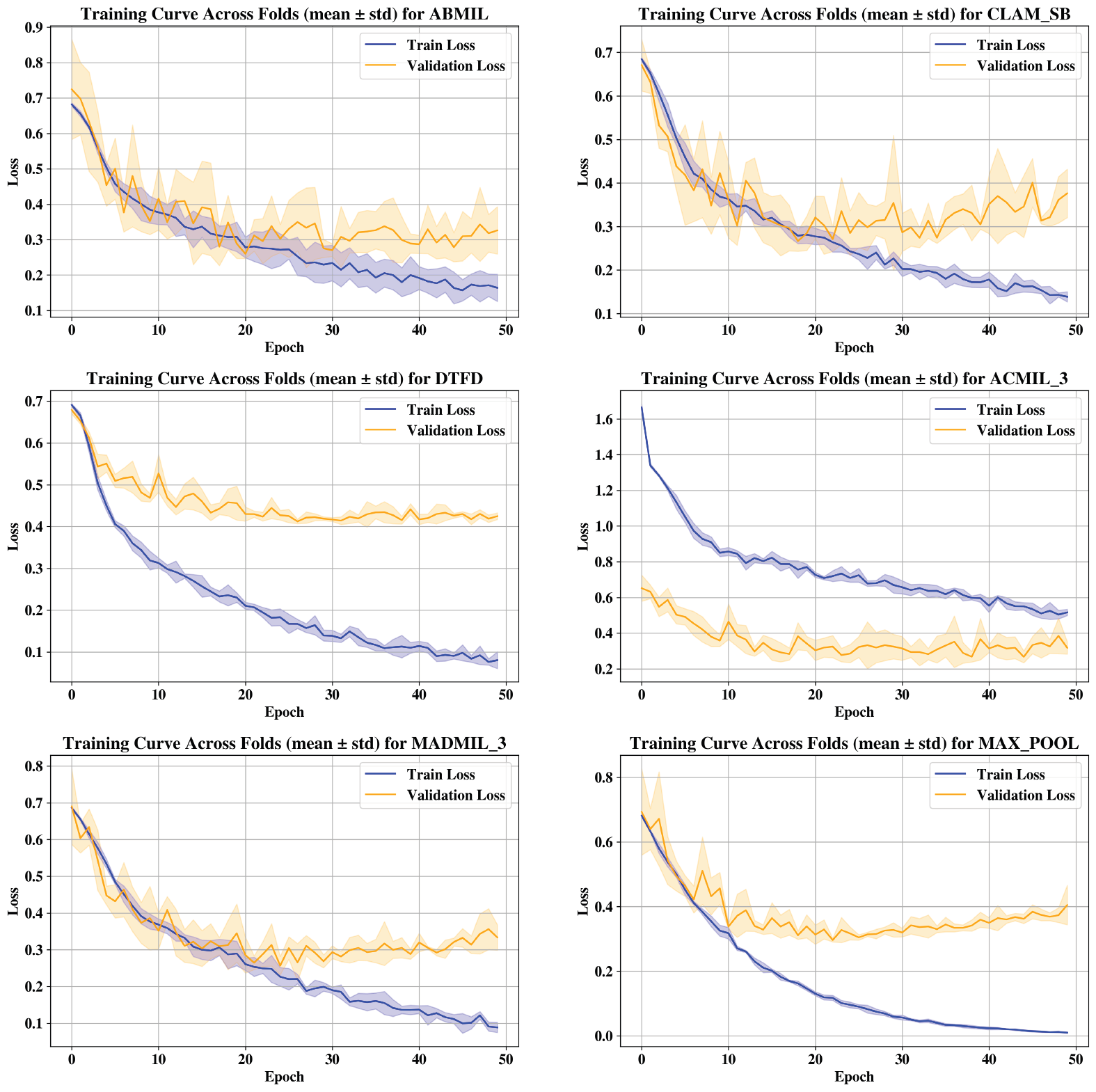

Supplement: S1 Fig — Each panel shows the training curve for one model on TCGA BRCA. (TIF) [file pone.0337261.s001.tif]

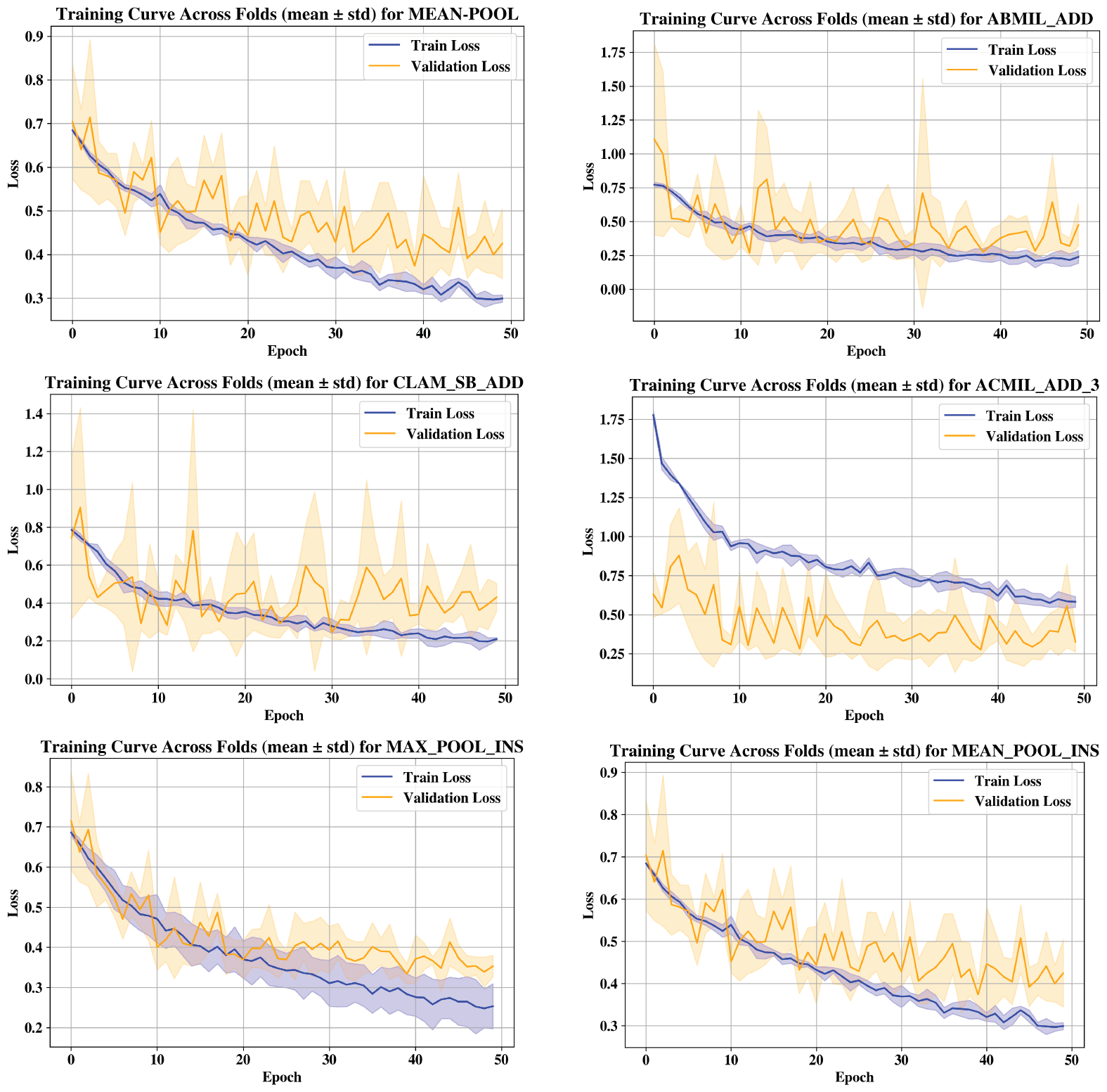

Supplement: S2 Fig — Each panel shows the training curve for one model on TCGA BRCA. (TIF) [file pone.0337261.s002.tif]
